# Comparative transcriptome analysis of vegetable soybean grain discloses genes essential for grain quality

**DOI:** 10.1186/s12870-024-05214-1

**Published:** 2024-06-03

**Authors:** Bin Wang, Yuanpeng Bu, Guwen Zhang, Na Liu, Zhijuan Feng, Yaming Gong

**Affiliations:** 1 Institute of Vegetables, Zhejiang Academy of Agricultural Sciences, 198, Shiqiao Rd, Hangzhou, 310021 Zhejiang China; 2 Key Laboratory of Vegetable Legumes Germplasm Enhancement and Molecular Breeding in Southern China of Ministry of Agriculture and Rural Affairs, 198, Shiqiao Rd, Hangzhou, 310021 Zhejiang China

**Keywords:** Vegetable soybean, Grain quality, Soluble sugar, Large grain, Transcriptome analysis

## Abstract

**Background:**

Vegetable soybean is an important vegetable crop in world. Seed size and soluble sugar content are considered crucial indicators of quality in vegetable soybean, and there is a lack of clarity on the molecular basis of grain quality in vegetable soybean.

**Results:**

In this context, we performed a comprehensive comparative transcriptome analysis of seeds between a high-sucrose content and large-grain variety (Zhenong 6, ZN6) and a low-sucrose content and small-grain variety (Williams 82, W82) at three developmental stages, i.e. stage R5 (Beginning Seed), stage R6 (Full Seed), and stage R7 (Beginning Maturity). The transcriptome analysis showed that 17,107 and 13,571 differentially expressed genes (DEGs) were identified in ZN6 at R6 (vs. R5) and R7 (vs. R6), respectively, whereas 16,203 and 16,032 were detected in W82. Gene expression pattern and DEGs functional enrichment proposed genotype-specific biological processes during seed development. The genes participating in soluble sugar biosynthesis such as *FKGP* were overexpressed in ZN6, whereas those responsible for lipid and protein metabolism such as *ALDH3* were more enhanced in W82, exhibiting different dry material accumulation between two genotypes. Furthermore, hormone-associated transcriptional factors involved in seed size regulation such as *BEH4* were overrepresented in ZN6, exhibiting different seed size regulation processes between two genotypes.

**Conclusions:**

Herein, we not only discovered the differential expression of genes encoding metabolic enzymes involved in seed composition, but also identified a type of hormone-associated transcriptional factors overexpressed in ZN6, which may regulate seed size and soluble content. This study provides new insights into the underlying causes of differences in the soybean metabolites and appearance, and suggests that genetic data can be used to improve its appearance and textural quality.

**Supplementary Information:**

The online version contains supplementary material available at 10.1186/s12870-024-05214-1.

## Background

Vegetable soybean (*Glycine max* L.), also called “edamame”, is harvested at full-seed development stage [1, 2]. Vegetable soybean has been extensively used for more than 2000 years in East Asian countries [3]. In recent years, acceptance of vegetable soybean-based food products has been reported in North and South America, Europe, and Africa [4]. Vegetable soybean has become a popular food all over the world because of their taste and nutritional value.

For vegetable soybean, grain quality has three characteristics, i.e. eating quality, appearance quality, and nutritional quality [5, 6]. The seeds of soybean are rich in protein, oil, and carbohydrates; as much as 47% of the carbohydrates are soluble sugars [7, 8]. Soluble sugar content is an important determinant of eating quality for vegetable soybean [9]. Carbohydrates in soybean determine the taste and quality of soyfoods [10]. Soybean soluble sugars can also be used as a functional factor or food additive to produce various health foods [10–13]. Vegetable soybeans are high sucrose content cultivars. Soluble sugar content in seeds increased during early developmental stage and reached a peak at full seed stage (R6), thereafter decreasing [14, 15]. Studies have shown that abundant genetic variations in total soluble sugar content of soybean germplasm provide abundant germplasm resources for breeding [16]. Soluble sugar content is a complex quality trait controlled by multiple genes and influenced by distinct environmental factors [17]. Until now, few QTLs associated with a soluble sugar content of soybean seeds were characterized. Metabolome and transcriptome analyses have been successfully employed to identify key gene regulatory networks of soluble sugar in soybean seeds [18]. Compared with grain soybean, the free amino acids, carbohydrates, sterols and flavonoids were increased in vegetable soybean. Combined with transcriptome analysis, multiple DEGs were identified, including starch and sucrose metabolism and sucrose transport pathway genes [19]. However, the specific regulatory mechanisms are still unclear.

Seed size is an important aspect of appearance quality for vegetable soybean [20]. It is generally believed that 100-grain dry weight of vegetable soybean is more than 20–30 g. Seed size is a complex trait controlled by many quantitative trait loci (QTL). Only a small subset of genes has been functionally characterized to be involved in soybean seed size regulation. *Seed thickness 1* (*ST1*), encoding a UDP-D-glucuronate 4-epimerase, affected seed thickness by influencing the pectin biosynthesis [21]. *Seed Thickness 05* (*GmST05*), encoding a transcriptional factor, positively controlled soybean seed thickness and size *via* regulating *GmSWEET10a* expression level [22]. A *phosphatase 2 C-1* (*PP2C-1*) allele from wild soybean ZYD7 contributed to the increase in seed size by modulating the brassinosteroid signaling pathway [23]. Furthermore, it has been found that there was an association between seed size and seed composition. *ST1*, *GmST05*, and *PP2C-1* regulated seed weight, simultaneously affecting oil content in soybean.

Recent studies suggested that vegetable soybeans were domesticated independently and clustered on a separate branch distinct from grain and wild soybean [24]. Vegetable soybeans exhibit large seeds and high soluble sugar content, because of domestication and breeding for improving soybean quality. Intrigued by these findings, we wondered that what factors simultaneously affected seed quality (seed size and soluble sugar content) in vegetable soybean. In this study, we characterized the grain quality of two genotypes, Zhenong6 (ZN6) and Williams82 (W82). ZN6, an outstanding commercial vegetable soybean cultivar in China, has large grain and high soluble sugar content. In contrast, the grain soybean cultivar, W82, exhibited precisely the opposite phenotype. Comparative transcriptome analysis was then employed for the seeds of the two genotypes to clarify two major issues: (i) what are the differences in the transcriptional regulation associated with different genotypes; (ii) what is the genetic basis for the different phenotypes of seed composition and size between the two genotypes. On the basis of bioinformatics analysis, it is expected that results of this study could elucidate the molecular mechanism underlying grain quality of vegetable soybean and provide a fundamental basis for genetic improvement of high-quality vegetable soybean.

## Results

### Significant grain size and seed composition differences between ZN6 and W82

**Fig. 1:**
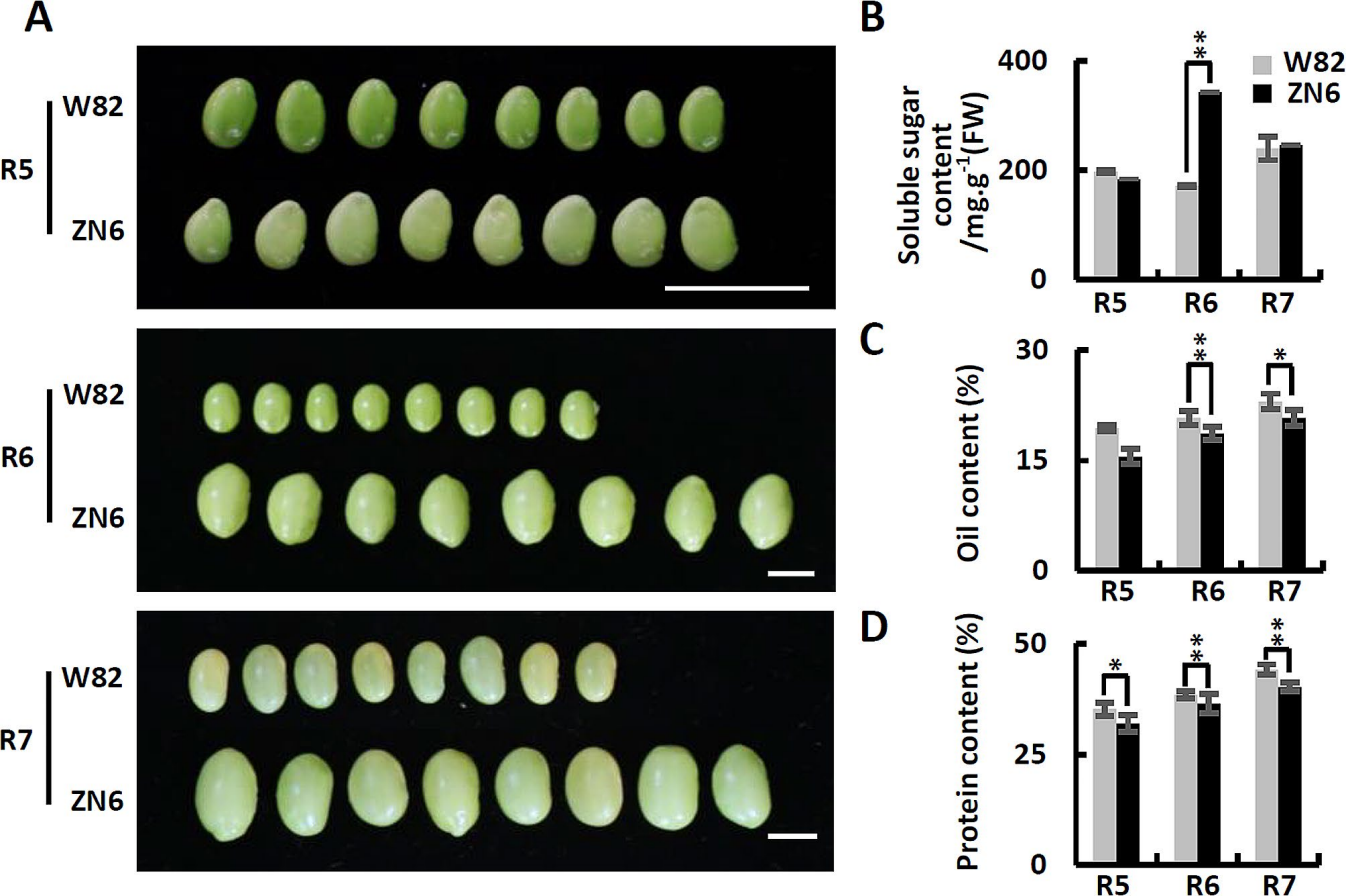
Phenotype differences between high-sucrose content and large-grain variety (Zhenong 6, ZN6) and low-sucrose content and small-grain variety (Williams 82, W82) at three developmental stages. (**A**) Visual appearance of ZN6 and W82 seeds at stage R5 (Beginning Seed), stage R6 (Full Seed), and stage R7 (Beginning Maturity). Scale bars = 1 cm. (**B**) Dynamic changes of soluble sugar content in ZN6 and W82 seeds at stage R5, stage R6, and stage R7. (**C**) Dynamic changes of protein content in ZN6 and W82 seeds at stage R5, stage R6, and stage R7. (**D**) Dynamic changes of lipid content in ZN6 and W82 seeds at stage R5, stage R6, and stage R7. The data are presented as the mean ± SE of three replicates. Asterisks indicate the significant differences between the two groups (Student’s t test, * *p* < 0.05; ** *p* < 0.01)

ZN6 is a vegetable soybean variety widely grown in southern China. Compared to grain soybean variety W82, ZN6 has a larger grain size (Fig. 1A; Fig. S1). At the R6 stage, the size of ZN6 seeds is approximately 2 times larger than that of W82 seeds. In addition, we found that there are also significant differences between the two varieties in the seed composition (Fig. 1B and D). Among different seed development stages, the soluble sugar content showed a general trend of increasing first and then decreasing in ZN6 and W82 (Fig. 1B). The highest point appeared around R6, at which time the soluble sugar content of ZN6 was always about two-fold higher than that of W82. With the development of seeds, we found that the oil content and the protein content increased gradually. The protein and oil content of W82 was always higher than that of ZN6 at the R5-R7 stage of seed development (Fig. 1C-D; Fig. S2). These results confirmed genotype-dependent differences in soybean appearance quality and nutritional quality, as shown in previous studies. Genetic stability of soybean seed composition and size implies differences in gene involvement between different varieties.

### Transcriptome profiles of the two genotypes during seed development

To identify the gene expression changes during grain development, we performed comparative transcriptomic analysis of W82 and ZN6 grains at R5, R6, and R7 stage. A total of 142,684,437 and 135,066,119 clean reads (47,603,993 for W82-R5, 49,560,619 for W82-R6, 45,519,825 for W82-R7, 47,796,749 for ZN6-R5, 42,681,775 for ZN6-R6, and 44,587,595 for ZN6-R7) were identified from the W82 and ZN6 transcriptome, respectively (Table 1; Fig. S3). An average of 96.72% of the reads were mapped to the soybean genome *Glycine max* Wm82.a4.v1 [25]. Using a cutoff of ≥ 2-fold change, a total of 16,203 and 17,107 DEGs were detected in W82 and ZN6 at R6 stage, respectively, as compared with those at R5 stage, whereas 16,032 and 13,571 genes were differentially expressed at R7 respectively, as compared with those at R6 stage. W82 exhibited large transcriptional changes during later stages of seed development (Fig. S4). According to previous research, the developing stages (stages R5 to R7) refer to the most active phase of grain filling [26, 27]. For legume, sugar significantly preferentially accumulated in seeds before stage R6. After stage R6, most of the reduced sucrose may be converted into oil or protein during seed development [26, 27]. The transcriptional changes at the R6-R7 stages of seed development may partially be related to higher concentrations of total protein and oil in W82 seeds. These results further confirmed that grain quality was genotype-dependent for vegetable soybean.

**Table 1:** Summary of transcriptome data by the Illumina platform

Sample	ZN6-R5	ZN6-R6	ZN6-R7	W82-R5	W82-R6	W82-R7	Sum/Ave
Raw reads	47,952,208	42,822,501	44,738,343	47,751,998	49,732,024	45,668,317	278,665,391
Raw bases	7,192,831,200	6,423,375,200	6,710,751,500	7,162,799,700	7,459,803,600	6,850,247,500	41,799,808,700
Q20 (%)	96.86	97.21	96.98	96.94	97.03	97.26	97.05
Q30 (%)	91.75	92.44	91.96	91.90	92.09	92.58	92.12
Clean reads	47,796,749	42,681,775	44,587,595	47,603,993	49,560,619	45,519,825	277,750,556
Clean bases	6,895,969,926	6,851,491,035	6,974,214,310	7,084,154,032	7,080,872,671	6,782,636,810	41,669,338,782
Q20 (%)	97.02	97.37	97.14	97.10	97.19	97.42	97.21
Q30 (%)	91.95	92.63	92.15	92.09	92.29	92.77	92.31
Mapped reads	46,032,705	41,073,613	42,827,007	46,093,739	47,568,359	43,938,133	267,533,556
Proportion (%)	96.41	96.84	96.34	96.99	96.81	96.91	96.72

### The transcriptome validation for DEGs by qRT-PCR

**Fig. 2:**
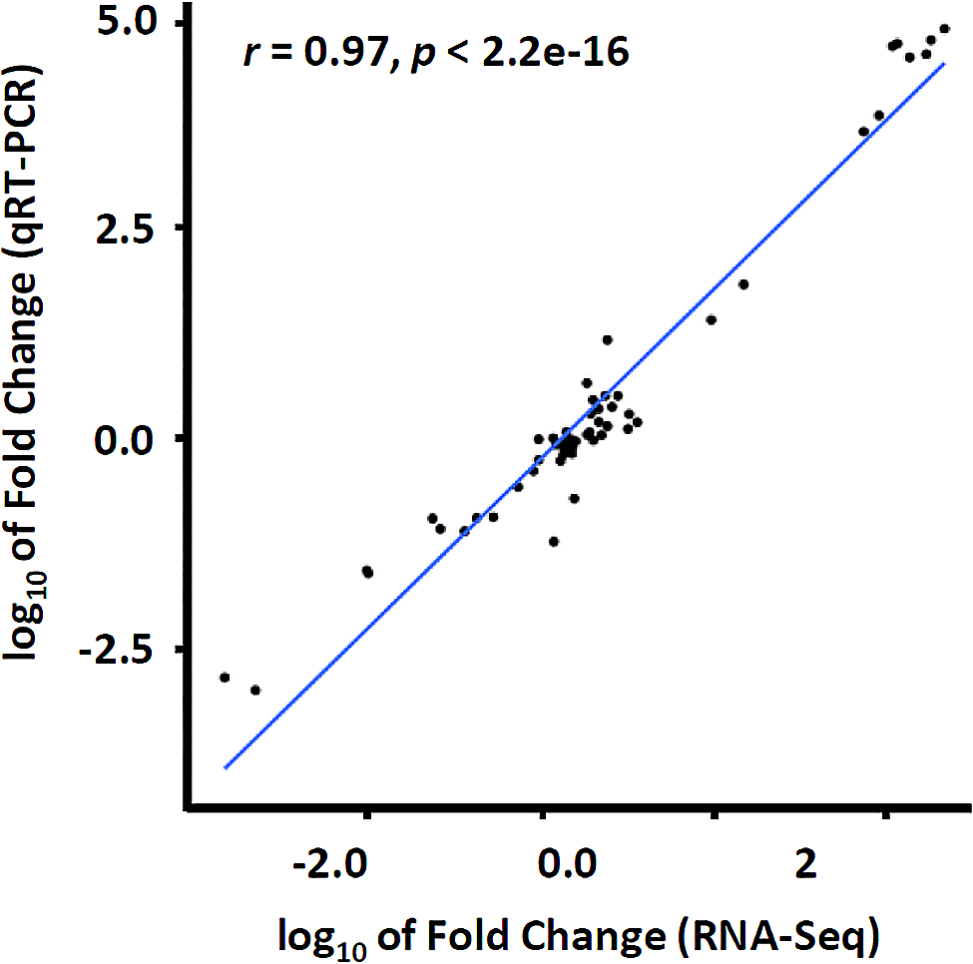
Correlation between qRT-PCR data and RNA-sequencing for the selected genes. Each point represents a value of fold change of expression level at R6 or R7 comparing with that at R5 or R6. Fold change values were transformed by log_10_

To validate these expression patterns of genes from the RNA sequencing (RNA-Seq) results, we randomly selected 8 genes for qRT-PCR analysis. The results showed a high level of consistency between the qRT-PCR and RNA-sequencing data (Fig. 2). For each gene, the expression count values from RNA sequencing data exhibited similar expression profile at all the three stages comparing with the results of qRT-PCR (Fig. S5). The results indicated that the RNA-Seq data of the present study were accurate and reliable.

### Gene expression pattern analysis and functional enrichment of DEGs

**Fig. 3:**
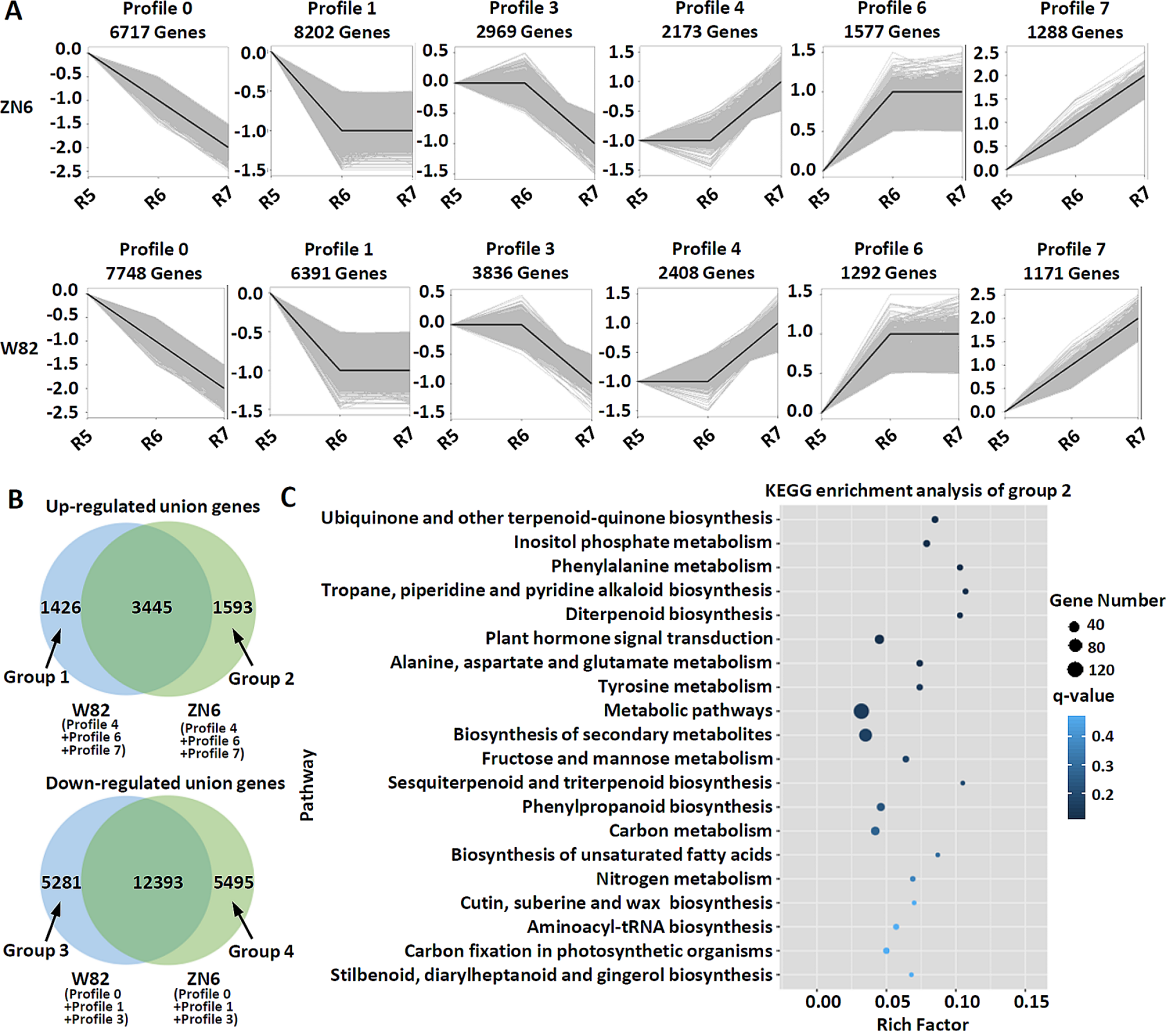
Patterns of gene expressions and KEGG enrichment across three time points in ZN6 and W82 seeds. (**A**) Patterns of gene expressions across three time points in ZN6 and W82 inferred by STEM analysis. In each frame, the black line represented the expression tendency of all the genes, while the light gray lines represented the expression pattern of each gene. (**B**) Venn diagram analysis of genes groups shows gene sets unique to W82 and ZN6. The profiles were clustered into four classes: ZN6 up-regulated genes group (Profile 4, profile 6, and profile 7 from ZN6), ZN6 down-regulated genes group (Profile 0, profile 1, and profile 3 from ZN6), W82 up-regulated genes group (Profile 4, profile 6, and profile 7 from W82), and W82 down-regulated genes group (Profile 0, profile 1, and profile 3 from W82). (**C**) KEGG enrichment analysis of group 2

Considering the differences in the genetic backgrounds of W82 and ZN6, we analyzed differences in gene expression pattern to find key genes that regulate grain quality [28, 29]. DEGs of each W82 and ZN6 at different time stages were clustered in eight profiles based on gene expression pattern (Fig. 3A). The 16 profiles were further divided into four classes: ZN6 up-regulated genes group (Profile 4, profile 6, and profile 7 from ZN6), ZN6 down-regulated genes group (Profile 0, profile 1, and profile 3 from ZN6), W82 up-regulated genes group (Profile 4, profile 6, and profile 7 from W82), and W82 down-regulated genes group (Profile 0, profile 1, and profile 3 from W82). To explore genes involved in grain quality in ZN6, we examined gene sets unique to W82 and ZN6 by performing clustering analysis with specific gene groups, respectively (Fig. 3B). A total of 1426 (Group 1 for W82) and 1593 (Group 2 for ZN6) genes were specifically upregulated during seed development (Fig. 3B; Table S2-S5). KEGG pathway enrichment analysis further revealed soluble sugar accumulation and seed size regulation (*p* < 0.05) were overrepresented simultaneously in ZN6 (Fig. 3C; Table S6). In contrast, genes involved in lipid and protein accumulation (*p* < 0.05) were significantly enriched in W82 (Fig. 3B; Fig. S6-S8; Table S6). These results suggested genotype-specific variation in genes and pathways regulating grain quality. The differentiation in gene expression patterns between the two genotypes was corresponding to their distinct seed composition accumulation and seed size regulation.

### Differential expression of genes involved in metabolic pathways contributed to seed composition variation in W82 and ZN6

**Fig. 4:**
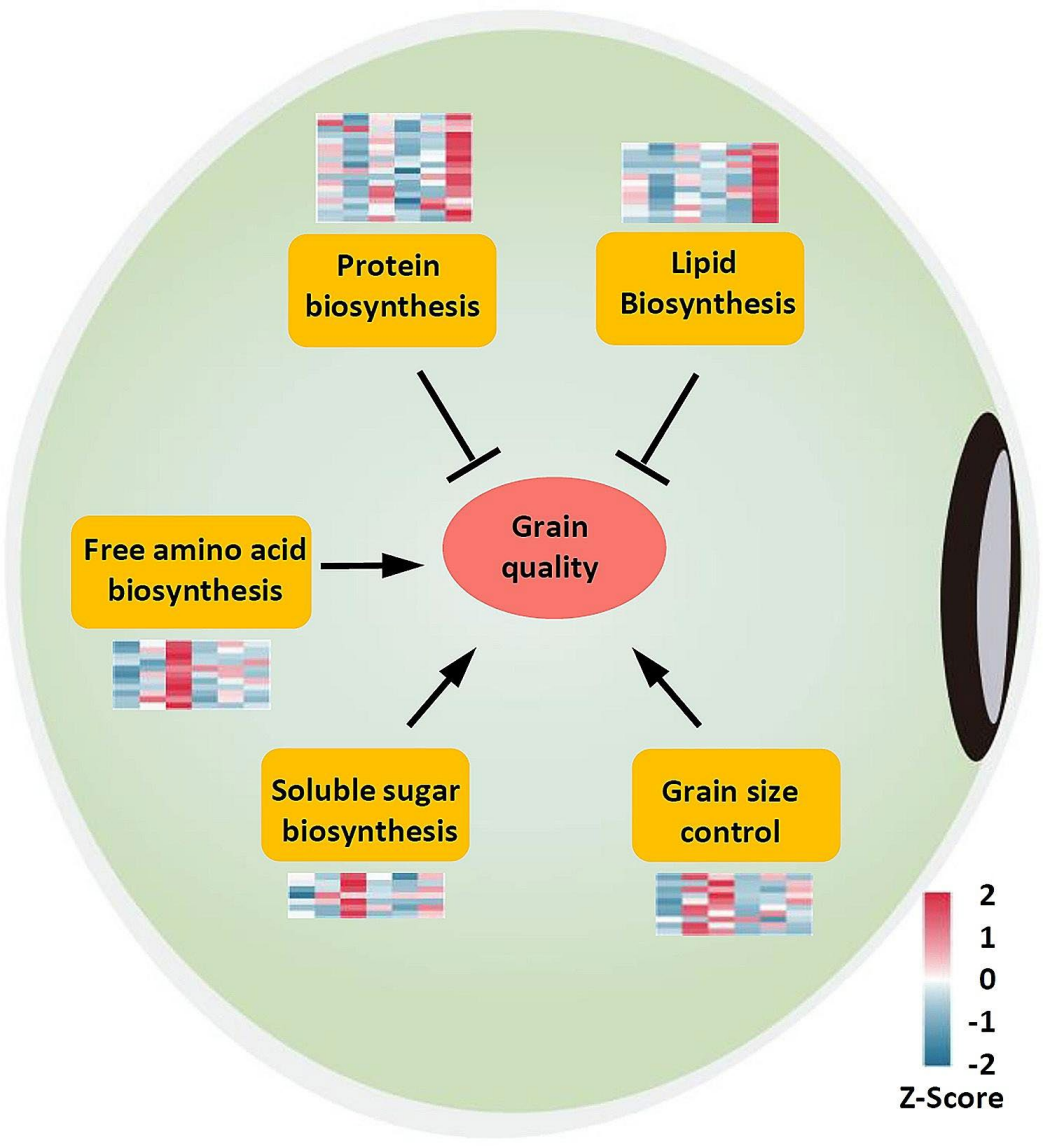
Transcriptional changes of genes responsible for grain quality formation in seeds of the two soybean genotypes. The metabolites biosynthesis pathway and grain size control responsible for grain quality are represented in orange boxes. The expression pattern of the genes involved in metabolites biosynthesis pathway and grain size control over the three time points in both two genotypes are shown beside each box. Each square represents a comparative pair. The squares from left to right represent: ZN6-R5, ZN6-R6, ZN6-R7, W82-R5, W82-R6, and W82-R7. The red, blue squares indicate their expression levels. Arrows indicate promotion and blockhead arrows inhibition

To uncover the genes responsible for the different nature of seed composition accumulation between W82 and ZN6, gene expression pattern and DEGs functional enrichment were performed. The activity of the enzymes in the metabolite synthesis were suggested to play the major role in genotype difference of seed composition accumulation [30]. Between the two genotypes, nine DEGs involved in monosaccharides biosynthesis were upregulated in ZN6 (Fig. 4; Table S6). 43 DEGs belonged to three GO terms involved in lipid and protein metabolism, which were upregulated in W82 (Fig. 4; Table S6). Besides these genes, another two DEGs encoding serine/threonine-protein kinase SRK2, which have also been proposed to be related to sucrose accumulation in seeds [31], were also identified in ZN6 up-regulated genes group (Fig. 4; Table S6). The enhanced expressions of these genes in W82 and ZN6 may thus contribute to change in metabolic flow direction, leading to significant variation in seed components.

In addition, 11 genes involved in free amino acids metabolism, such as glutamate synthase and homogentisate 1,2-dioxygenase, were overexpressed in ZN6 (Fig. 4; Table S6). It is known that free amino acids are closely related to flavor and can be used as indicators of quality and freshness [32, 33]. The overexpression of the genes responsible for free amino acids metabolism was thought to result in the unique flavor of ZN6.

### Genes involved in hormone signal transduction acted as seed size control in ZN6

Besides soluble sugar and free amino acids biosynthesis, ZN6 also employed specific regulatory mechanism to improve grain quality. Among the up-regulated genes in ZN6, hormone-associated transcriptional factors genes were found to be overrepresented, most of which are involved in auxin, brassinosteroid, and cytokinin signal transduction (Fig. 4; Table S6). Multiple studies have demonstrated that auxin, brassinosteroid, and cytokinin regulate seed size in plants.

To confirm that sugar metabolism and grain size control together contributed to grain quality traits in vegetable soybean, we analyzed expression of the selected quality-associated genes in different soybean varieties at stage R6 (Fig. S8A-C). The results showed that genes involved in sugar metabolism and grain size control were overexpressed in vegetable soybean varieties XNQY and ZNQF. In contrast, protein and lipid metabolism genes were upregulated in grain soybean varieties TL and HC6. These results suggested that there are conserved regulatory pathways of grain quality between vegetable soybean varieties.

## Discussion

Vegetable soybean has become a popular food all over the world because of their taste and nutritional value. According to these previous studies [24], vegetable soybean was domesticated independently. A cluster analysis found that vegetable soybean is clearly clustered on a separate branch distinct from grain and wild soybean. Many useful quality traits have been bred into vegetable soybean cultivars, such as large seeds and high soluble sugar content. In contrast, for grain soybean, high protein content or oil content were selected. Seed size and soluble content are two important aspects of vegetable soybean quality. Harrigan et al. (2015) selected nine soybean varieties and performed metabolomics analysis of mature grains. They found that glucose and sucrose showed large variation across varieties [34]. We characterized the grain quality of vegetable soybean cultivar ZN6. Compared to grain soybean variety W82, ZN6 has a larger grain size (Fig. 1A; Fig. S1). In addition, we found that ZN6 contained relatively high levels of soluble sugar (Fig. 1B). On the contrary, W82 contained relatively high levels of protein and oil (Fig. 1C-D; Fig. S2). This, combined with evolutionary analysis, allowed us to speculate that the grain quality in vegetable soybean is genotype-dependent.

Seed composition is a complex quality trait that is controlled by multiple genes and influenced by distinct environmental factors [17]. Until now, few QTLs and genes associated with soybean seed composition were reported. Previous studies also discovered some regulatory genes for soluble sugar accumulation in vegetable soybeans using metabolome and transcriptome [18, 35]. Compared with grain soybean, the free amino acids, carbohydrates, sterols, and flavonoids were increased in vegetable soybean. Combined with transcriptome analysis, multiple DEGs were identified, including starch and sucrose metabolism and sucrose transport pathway genes [19]. In this study, we found that the genes participating in soluble sugar biosynthesis were overexpressed in ZN6 (Fig. 4; Table S6), whereas those responsible for lipid and protein metabolism were more enhanced in W82, exhibiting different dry material accumulation between two genotypes.

The synergistic interaction between maternal and zygotic tissues determines the final seed size [36]. Recent advances have identified several signaling pathways that control seed size, including or involving the ubiquitin-proteasome pathway, G-protein signaling, mitogen-activated protein kinase (MAPK) signaling, phytohormone perception and homeostasis, and some transcriptional regulators. Brassinosteroids (BRs) and auxin are two major hormones involved in maternal control of seed size. The BR biosynthesis mutants and BR insensitive mutants produced short seeds, suggesting that BR promotes seed growth [34, 37–42]. Auxin homeostasis can influence seed size by regulating endosperm development. Evidence from Arabidopsis and rice suggested the roles of auxin signaling in seed size control [40, 43–50]. In this study, we found that hormone-associated transcriptional factors genes were found to be overrepresented in ZN6, most of which are involved in auxin, brassinosteroid, and cytokinin signal transduction (Fig. 4; Table S6). Previous studies have found that there is an association between seed size and seed composition. *ST1*, *GmST05*, and *PP2C-1* regulated seed weight, simultaneously affecting oil content in soybean [19, 21–23]. Thus, we speculate that hormone-associated transcriptional factors genes in ZN6 regulated seed size and affected the accumulation of soluble sugar.

## Conclusion

In summary, there are two important conclusions from this work: (i) many genes related to soluble sugar, lipid, and protein metabolism were differentially expressed between W82 and ZN6 during seed development; (ii) hormone-associated transcriptional factors genes overexpressed in ZN6 regulated seed size and affected the accumulation of soluble sugar (Fig. 4). This study has greatly enhanced our knowledge of the genetic regulatory network of grain quality for vegetable soybean, providing important clues for molecular assisted screening and breeding of high-quality cultivars for vegetable soybean.

## Materials and methods

### Plant material and sampling

Two identified soybean genotypes, a high-sucrose content and large-grain variety (Zhenong 6, ZN6) and a low-sucrose content and small-grain variety (Williams 82, W82), were used for transcriptome analysis in this study. Soybean plants were cultivated at the farmland of Zhejiang Academy of Agricultural Sciences (29°27′N, 120°23′E) in the summers of 2020 and 2021. All plants were subjected to the same field management during the entire growth period. According to previous research [26, 27], about forty seed samples from three plants of two genotype (ZN6 and W82) were collected for RNA sequencing and grain composition measurement with three biological replicates at R5, R6, and R7 stages, respectively. Stage R5 (Beginning seed) is seed filling phase; total pod weight peaks at stage R6 (Full seed); Seeds and pods begin to lose green color at stage R7 (Beginning maturity).

### Determination of soluble sugar content, oil content, and protein content

Three plants of each genotype as biological replicates were used for measurement of soluble sugar content, oil content, and protein content. Total soluble sugar content was measured using the Plant Soluble Sugar Content Assay Kit (Solarbio, China). The operation methods are strictly in accordance with the instructions. Soluble sugar content was expressed on a fresh weight basis as mg/g (FW). The oil content was quantified using the Soxhlet extraction method [51]. The protein content of seeds was determined by Kjeldahl analysis [52]. The total nitrogen content was converted to protein content by using a conversion factor (5.64 for soybean) [52].

### Identification of differentially expressed genes

For RNA extraction, about forty seed samples from three plants of two genotype (ZN6 and W82) were collected with three biological replicates at R5, R6, and R7 stages, respectively. mRNA was enriched by hybridization to oligo(dT) beads. The cDNA library was constructed with NEBNext Ultra RNA Library Prep Kit for Illumina (New England Biolabs, USA), and Sequencing was performed on a NovaSeq6000 platform (Illumina) by Gene Denovo Biotechnology Co. (China).

Reads from RNA sequencing were mapped to the soybean reference genome (*Glycine max* Wm82.a4.v1). Gene abundances were quantified using RSEM software. Differentially expressed genes (DEGs) were identified by DESeq2 (version 1.12.4). To agglomerate gene patterns along seeds development stages, Short Time-course Expression Miner (STEM) algorithm was used [53]. All the DEGs were clustered into eight expression profiles.

### Functional annotation and KEGG classification

All expressed genes were compared against various databases for functional annotation, including Clusters of Orthologous Groups of proteins database (COG), Kyoto Encyclopedia of Genes and Genomes (KEGG), NCBI nonredundant protein database (Nr), and Swiss-Prot database, by BLASTX searches with an e-value cutoff of 1e-5 in Blast2GO. Only the annotation with the highest score was used for each protein sequence. For each KEGG pathway, the numbers of up- and down-regulated genes of each genotype were compared to the reference set by Fisher’s exact test to find out the pathways enriched with up and down-regulated genes. KEGG enrichment analysis was also carried out for all the eight gene expression profiles.

### Gene expression validation

Eight genes from RNA sequencing data were randomly selected for validation by quantitative real-time PCR (qRT-PCR). Seed samples (ZN6 and W82) were collected with three biological replicates at R5, R6, and R7 stage, respectively. Total RNA was extracted from three biological replicates using an RNA isolation kit (Thermo Fisher Scientific, USA). First strand cDNA was prepared using TransScript® One-Step gDNA Removal and cDNA Synthesis SuperMix (Transgen, China). Gene-specific primers for qPCR were designed using Primer3 Web site (Table S1). The soybean *GmActin* gene (*Glyma.18G290800*) was used as the internal reference gene (Table S1). RT-PCR was performed with ChamQ SYBR qPCR Master Mix (Vazyme, China) and reactions were run on CFX96 Touch Real-Time PCR Detection System (Bio-Rad, USA). Three technical replicates were performed. Linear regression analysis was performed to evaluate the correlation of fold change data between qRT-PCR and RNA sequencing using R package (version 3.1.3).

### Statistical analysis

The RNA-seq analysis was described as above, and Student’s t-tests and Tukey test were performed using Excel and SPSS, respectively.

### Electronic supplementary material

Below is the link to the electronic supplementary material.

Supplementary Material 1

Supplementary Material 2

## Data Availability

The raw sequence data reported in this paper have been deposited in the Genome Sequence Archive (Genomics, Proteomics & Bioinformatics 2021) in National Genomics Data Center, China National Center for Bioinformation / Beijing Institute of Genomics, Chinese Academy of Sciences (GSA: CRA010668).
