# Supplementary material for: Comparative transcriptome analysis of vegetable soybean grain discloses genes essential for grain quality
Source: BMC Plant Biol. 2024 Jun 3;24:491. doi: 10.1186/s12870-024-05214-1 (PMC11145879; doi:10.1186/s12870-024-05214-1)

**Supplementary data**

**Figure S1.** Seed length of ZN6 and W82 at stage R5, R6, and R7. The data are presented as the mean ± SE of three replicates. Asterisks indicate the significant differences between the two groups (Student’s t test, * p < 0.05; ** p < 0.01).

**Figure S2.** Analysis of protein content in ZN6 and W82 seeds at stage R5, stage R6, and stage R7 using BCA method.

**Figure S3.** A heat map of the differentially expressed genes identified across three time points in ZN6 and W82 seeds.

**Figure S4.** The gene numbers of significantly different expression among samples.

**Figure S5.** Expression of the selected genes inferred by RNA sequencing and qRT-PCR.

**Figure S6.** KEGG enrichment analysis of group 1 (Fig. 3B).

**Figure S7.** KEGG enrichment analysis of group 3 (Fig. 3B).

**Figure S8.** KEGG enrichment analysis of group 4 (Fig. 3B).

**Figure S9.** Conserved regulatory pathways of grain quality between vegetable soybean varieties. **(A)** Visual appearance of grain soybean varieties (TL and HC6) and vegetable soybean varieties (XNQY and ZNQF) seeds at stage R6. Scale bars=1 cm. **(B)** Seed length of TL, HC6, XNQY, and ZNQF at stage R6. The data are presented as the mean ± SE of three biological replicates. Different letters at the top of each column indicate a significant difference at p < 0.05 determined by the Tukey test. **(C)** Expression analysis of the selected quality-associated genes in different soybean varieties at stage R6.

**Figure S1**

**
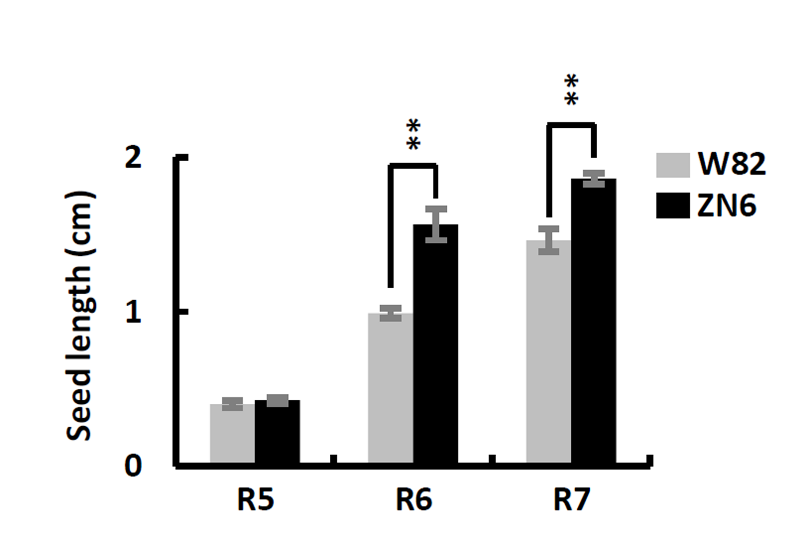
**

**Figure S2**

**
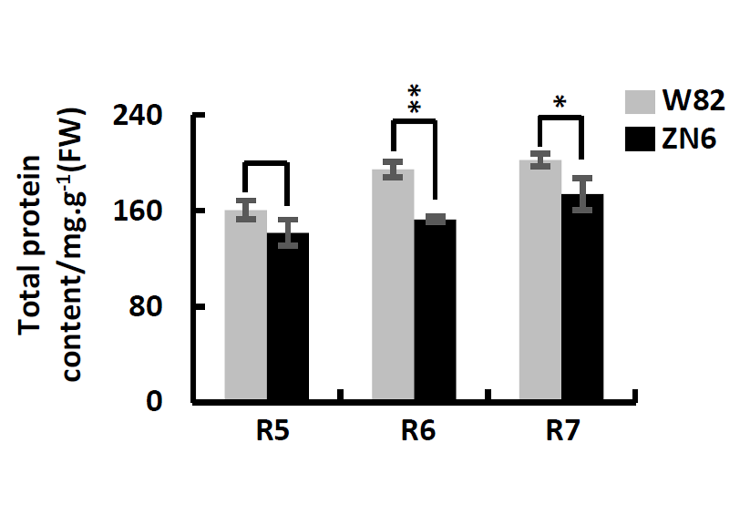
**

**Figure S3**


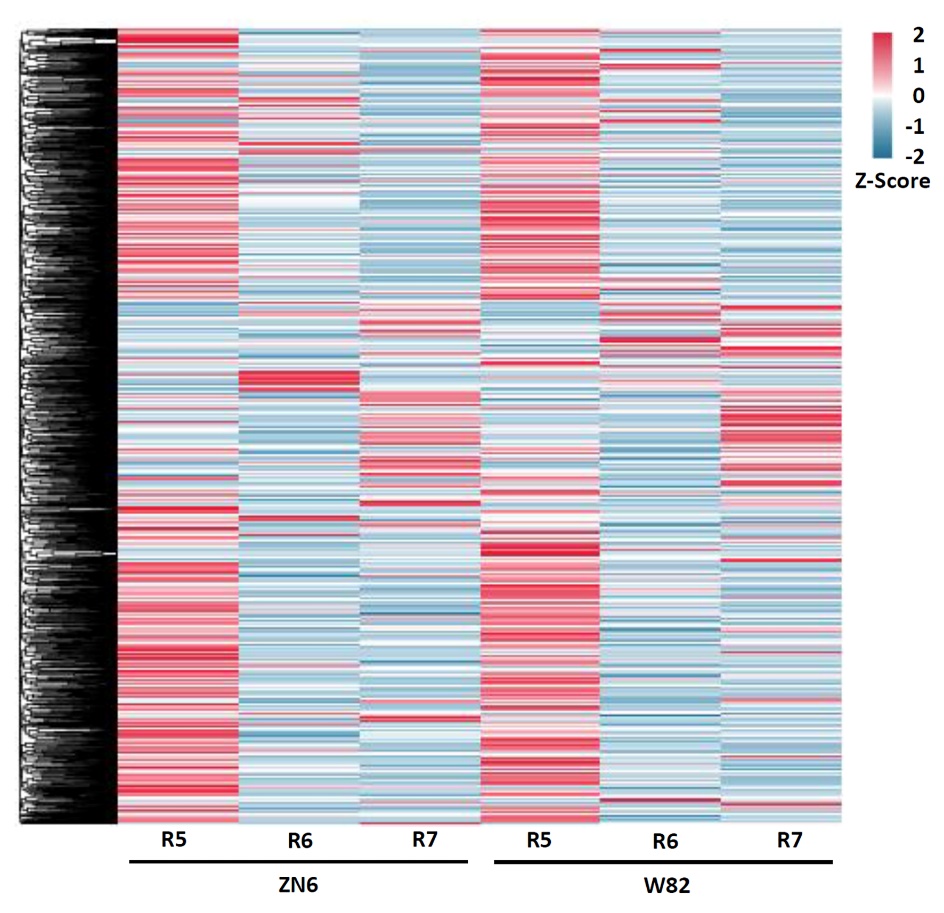


**Figure S4**


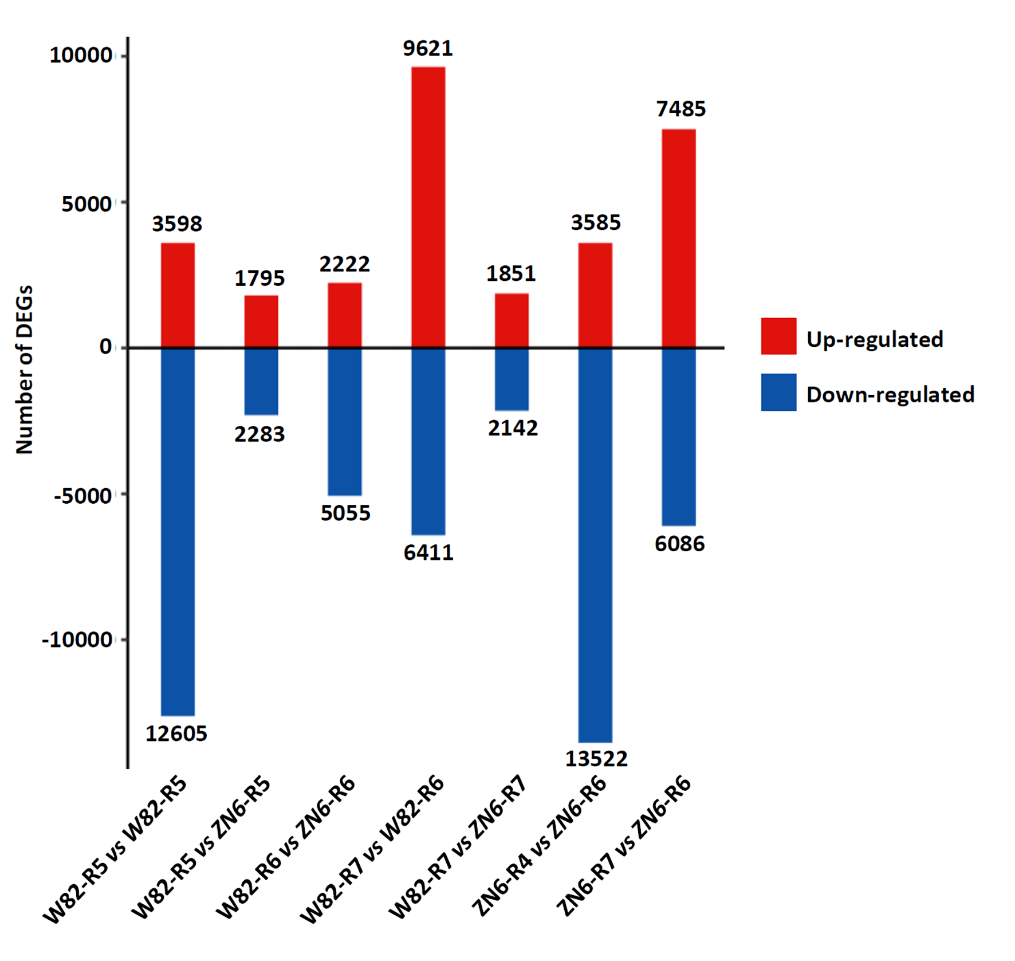


**Figure S5**


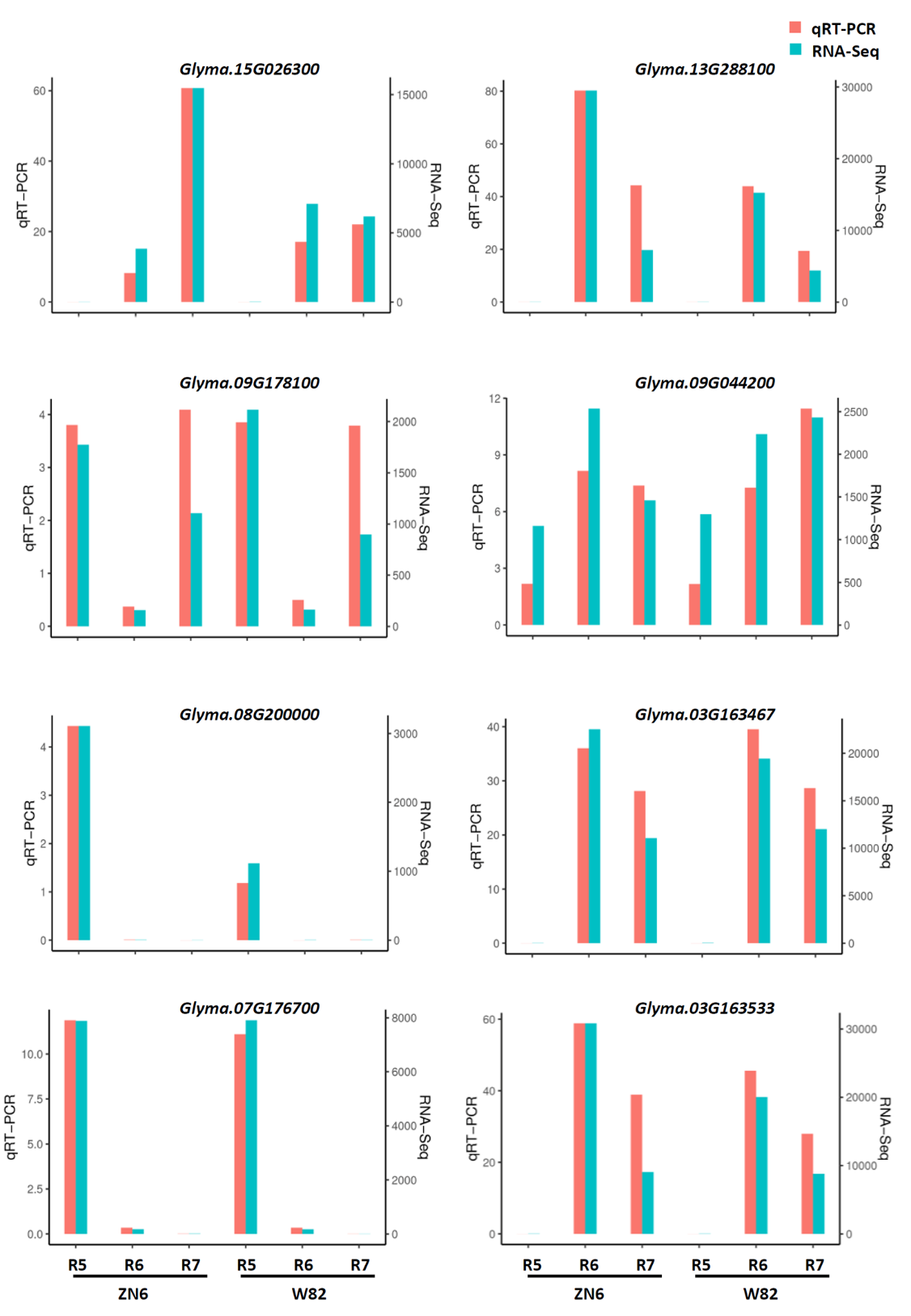


**Figure S6**


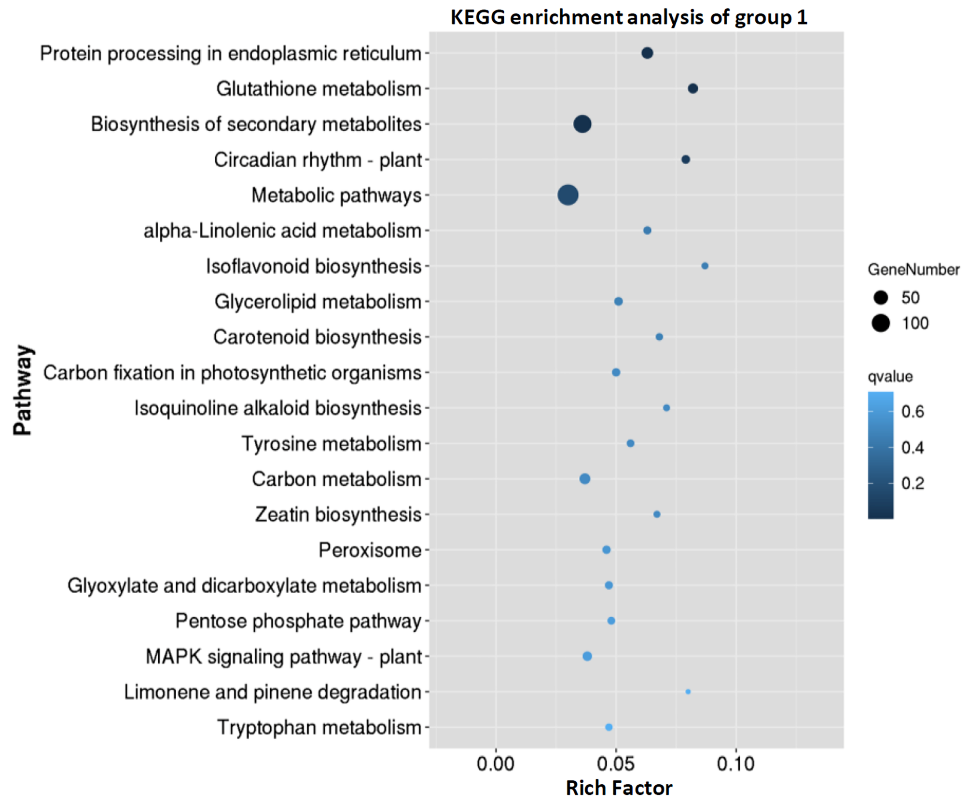


**Figure S7**


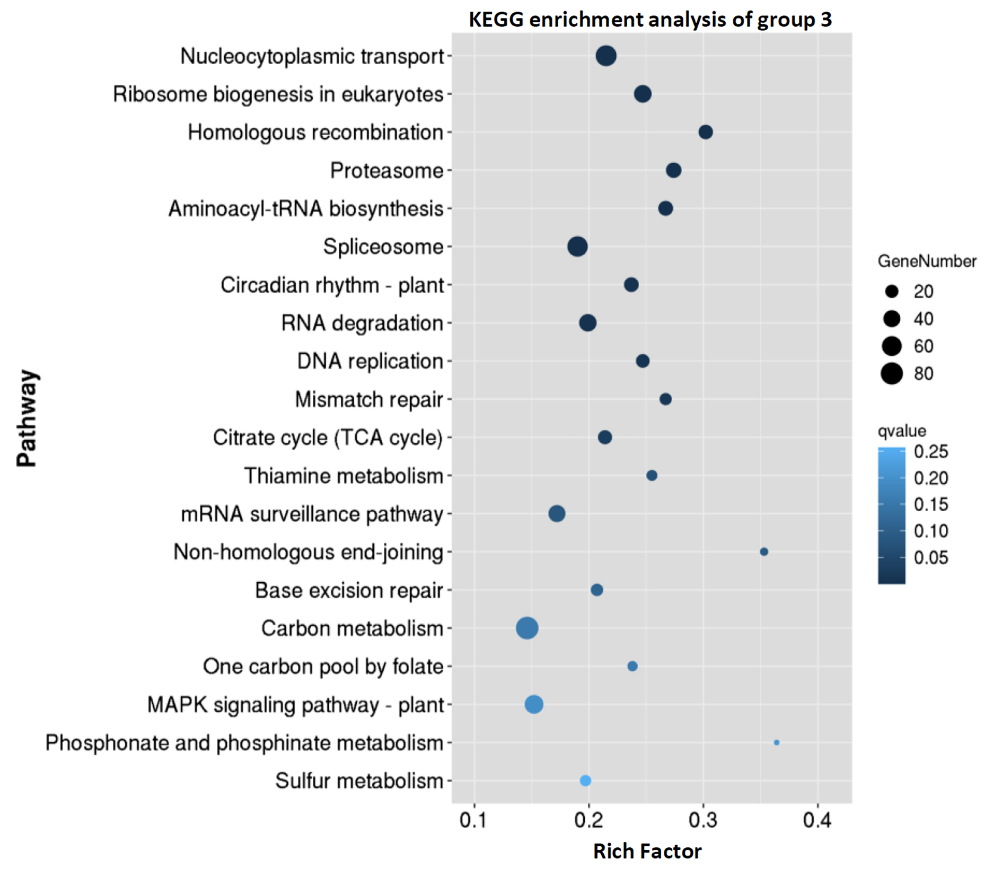


**Figure S8**


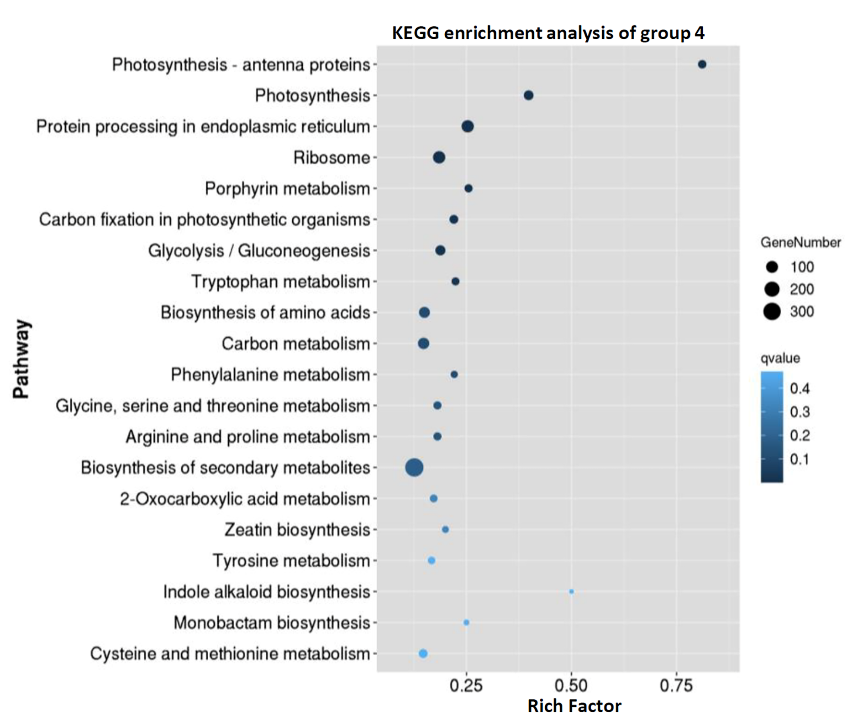


**Figure S9**


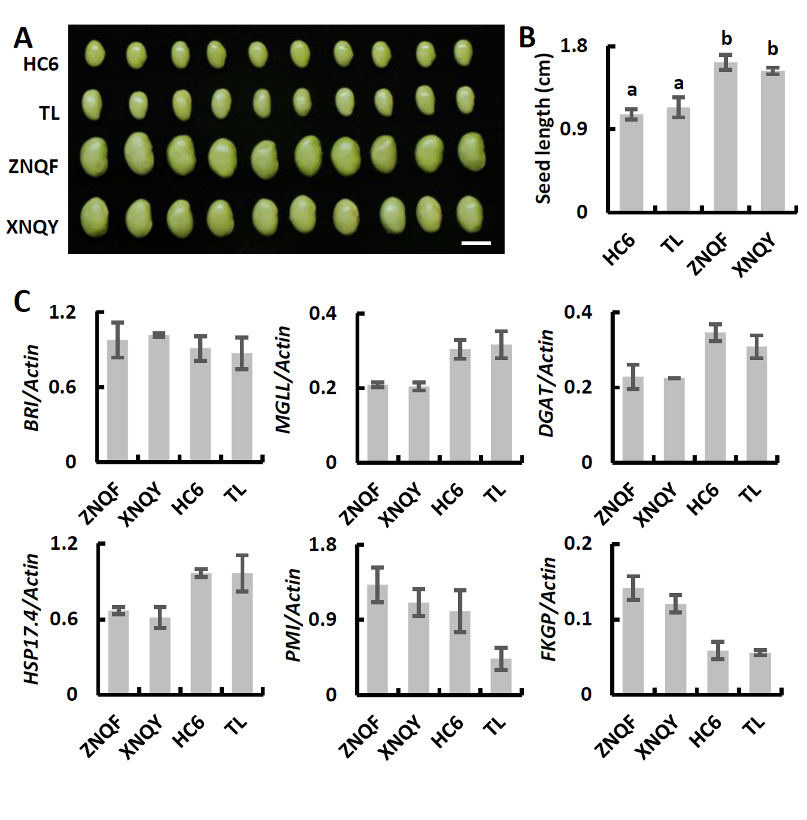

Supplement: Supplementary file 2 — Supplementary Material 2 [file 12870_2024_5214_MOESM2_ESM.docx]
